# *Listeria monocytogenes* virulence factor secretion: don't leave the cell without a chaperone

**DOI:** 10.3389/fcimb.2014.00013

**Published:** 2014-02-12

**Authors:** Laty A. Cahoon, Nancy E. Freitag

**Affiliations:** Department of Microbiology and Immunology, University of Illinois at ChicagoChicago, IL, USA

**Keywords:** PrsA, PrsA2, bacterial pathogenesis, PrfA, PPIase, foldase, LLO, cell wall

## Abstract

In Gram-positive bacteria, the secretion of proteins requires translocation of polypeptides across the bacterial membrane into the highly charged environment of the membrane-cell wall interface. Here, proteins must be folded and often further delivered across the matrix of the cell wall. While many aspects of protein secretion have been well studied in Gram-negative bacteria which possess both an inner and outer membrane, generally less attention has been given to the mechanics of protein secretion across the single cell membrane of Gram-positive bacteria. In this review, we focus on the role of a post-translocation secretion chaperone in *Listeria monocytogenes* known as PrsA2, and compare what is known regarding PrsA2 with PrsA homologs in other Gram-positive bacteria. PrsA2 is a member of a family of membrane-associated lipoproteins that contribute to the folding and stability of secreted proteins as they cross the bacterial membrane. PrsA2 contributes to the integrity of the *L. monocytogenes* cell wall as well as swimming motility and bacterial resistance to osmotic stress; however its most critical role may be its requirement for *L. monocytogenes* virulence and viability within host cells. A better understanding of the role of PrsA2 and PrsA-like homologs will provide insight into the dynamics of protein folding and stability in Gram-positive bacteria and may result in new strategies for optimizing protein secretion as well as inhibiting the production of virulence factors.

## Introduction

Bacteria are generally highly adaptable creatures that must interface with their varied environments to acquire nutrients, mitigate stress conditions, establish a replication niche, and avoid or eliminate undesirables, such as immune effector cells bent on bacterial destruction. Bacterial secreted proteins provide both structural and enzymatic functions that facilitate bacterial adaptation to environmental changes; following synthesis, these proteins must be translocated across the bacterial cell membrane and properly folded so as to carry out their functional roles. While considerable attention has been given to the process of protein secretion across the inner and outer membranes of Gram-negative bacteria, less attention has been generally focused on protein translocation across the single cell membrane of Gram-positive bacteria, which also possess a formidable cell wall. Gram-positive bacteria appear to have evolved protein secretion strategies that are both similar and distinct from those of their Gram-negative cousins.

*Listeria monocytogenes* is a Gram-positive facultative intracellular bacterial pathogen that has served as a model organism for studies focused on areas that include immunology, host-pathogen interactions, cell biology, and mechanisms by which environmental microbes become human pathogens (Cossart, 2011; Witte et al., 2012; Xayarath and Freitag, 2012). It has recently become apparent that *L. monocytogenes* is also an excellent model system for the investigation of Gram-positive protein secretion and the associated role of a critical post-translocation secretion chaperone known as PrsA2 (Alonzo et al., 2009, 2011; Zemansky et al., 2009; Alonzo and Freitag, 2010; Forster et al., 2011). PrsA2 is a member of a family of membrane-associated lipoproteins that contribute to the folding and stability of secreted proteins as they cross the bacterial membrane and enter the highly charged environment of the membrane-cell wall interface (Forster and Marquis, 2012). In this review, we describe the role of PrsA2 in *L. monocytogenes* protein secretion and compare what is known about *L. monocytogenes* PrsA2 with homologs in other Gram-positive bacterial species. Evidence indicates that when newly synthesized proteins foray into the challenging social scene of the membrane-cell wall interface, it is clearly advantageous to be properly chaperoned.

## Identification of PrsA2 (and PrsA1) as post-translocation secretion chaperones in *L. monocytogenes*

*L. monocytogenes* is considered an environmental pathogen as it is capable of life as a saprophyte in the outside environment while also maintaining the ability to invade and replicate within mammalian cells (Xayarath and Freitag, 2012). As part of the transition between life in the outside environment to life within the cytosol, *L. monocytogenes* increases the expression of secreted virulence factors that facilitate intracellular survival by promoting cell entry, bacterial escape from host vacuoles, replication within the cytosol, and spread to adjacent cells (Shetron-Rama et al., 2003; Mueller and Freitag, 2005; Port and Freitag, 2007; Alonzo and Freitag, 2010; de Las Heras et al., 2011; Mostowy and Cossart, 2012). The expression of many of the secreted virulence gene products required for bacterial survival within the host is regulated by the transcriptional activator PrfA (positive regulatory factor A) (Leimeister-Wachter et al., 1990; Mengaud et al., 1991; Freitag et al., 1992). Experimental evidence suggests that PrfA becomes highly activated upon bacterial entry into host cells, thereby leading to large increases in the synthesis and secretion of proteins that promote *L. monocytogenes* intracellular survival and replication. This substantial increase in virulence factor translocation across the bacterial membrane is likely to correspondingly require an increase in the activity of proteins directing protein secretion and folding.

*L. monocytogenes prsA2*, encoding a post-translocation molecular chaperone, was first identified via transcriptome analysis as a gene whose expression increased as a result of PrfA activation (Chatterjee et al., 2006) and which contained a putative PrfA binding site within its promoter region located 206 base pairs upstream from its translation start codon (Milohanic et al., 2003). Subsequent proteomic analysis of *L. monocytogenes* secreted proteins revealed that PrsA2 levels were increased in mutant strains containing constitutively activated PrfA (PrfA^*^) (Port and Freitag, 2007). Later, strains containing transposon insertions within *prsA2* were identified in a genetic screen designed to identify *L. monocytogenes* mutants with reduced hemolytic activity (Zemansky et al., 2009). While the presence of a PrfA binding site may enhance *prsA2* expression upon PrfA activation, this PrfA binding site is not essential for pathogenesis (Zemansky et al., 2009). Since its initial identification, *L. monocytogenes* PrsA2 and its homolog PrsA1 have been characterized for their roles in protein secretion and pathogenesis (Alonzo et al., 2009, 2011; Zemansky et al., 2009; Alonzo and Freitag, 2010; Forster et al., 2011).

*L. monocytogenes* PrsA2 contributes to multiple facets of bacterial pathogenesis and is essential for virulence (Alonzo et al., 2009; Zemansky et al., 2009), reflecting the apparent requirement of PrsA2 for the folding and secretion of a number of proteins (Alonzo and Freitag, 2010). PrsA1, in contrast, while sharing 75% amino acid similarity and 58% identity with PrsA2, makes no detectable contribution to *L. monocytogenes* pathogenesis (Alonzo et al., 2009). *L. monocytogenes* mutants lacking PrsA2 exhibit decreased hemolytic and phospholipase activity, and are defective for cell-to-cell spread (Alonzo et al., 2009; Zemansky et al., 2009; Forster et al., 2011). In mouse models of infection, *L. monocytogenes* strains lacking PrsA2 have at least 100,000-fold fewer colony forming units recovered from the livers and spleens of infected animals in comparison to animals infected with the wild-type strain (Alonzo et al., 2009; Zemansky et al., 2009). These data indicate that *L. monocytogenes* PrsA2 has been adapted for aspects of protein secretion that are critical for *L. monocytogenes* pathogenesis, whereas the function of PrsA1 remains to be identified. Neither *prsA1* nor *prsA2* is required for bacterial growth in broth culture as both genes can be deleted without significant effects on growth (Alonzo and Freitag, 2010).

## A family resemblance: comparison of *L. monocytogenes* PrsA2 with homologs in other gram-positive bacteria

**Figure 1:**
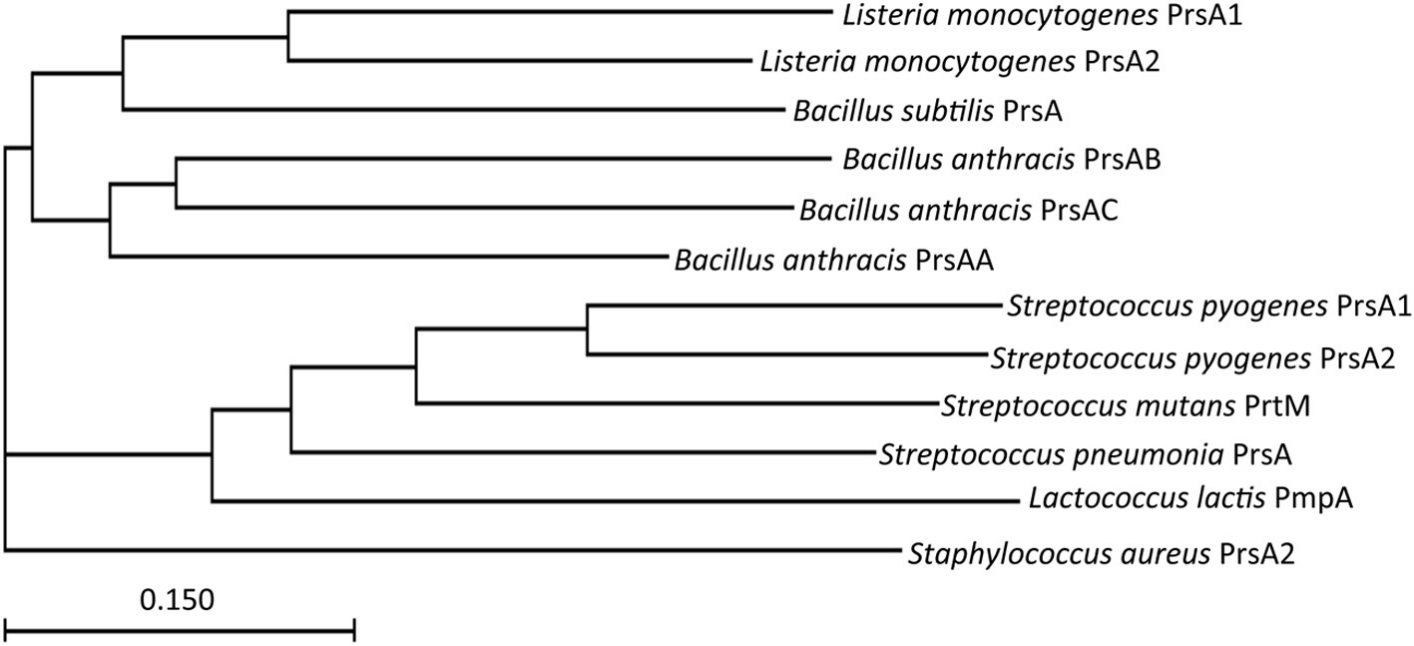
**The evolutionary relationship of PrsA homologs from Gram positive bacteria**. Shown is a phylogenetic tree of PrsA homologs from *Listeria monocytogenes* and other Gram positive bacteria created by CLUSTAL analysis. The scale bar indicates 1 substitution for every 10 nucleic acid residues.

**Figure 2:**
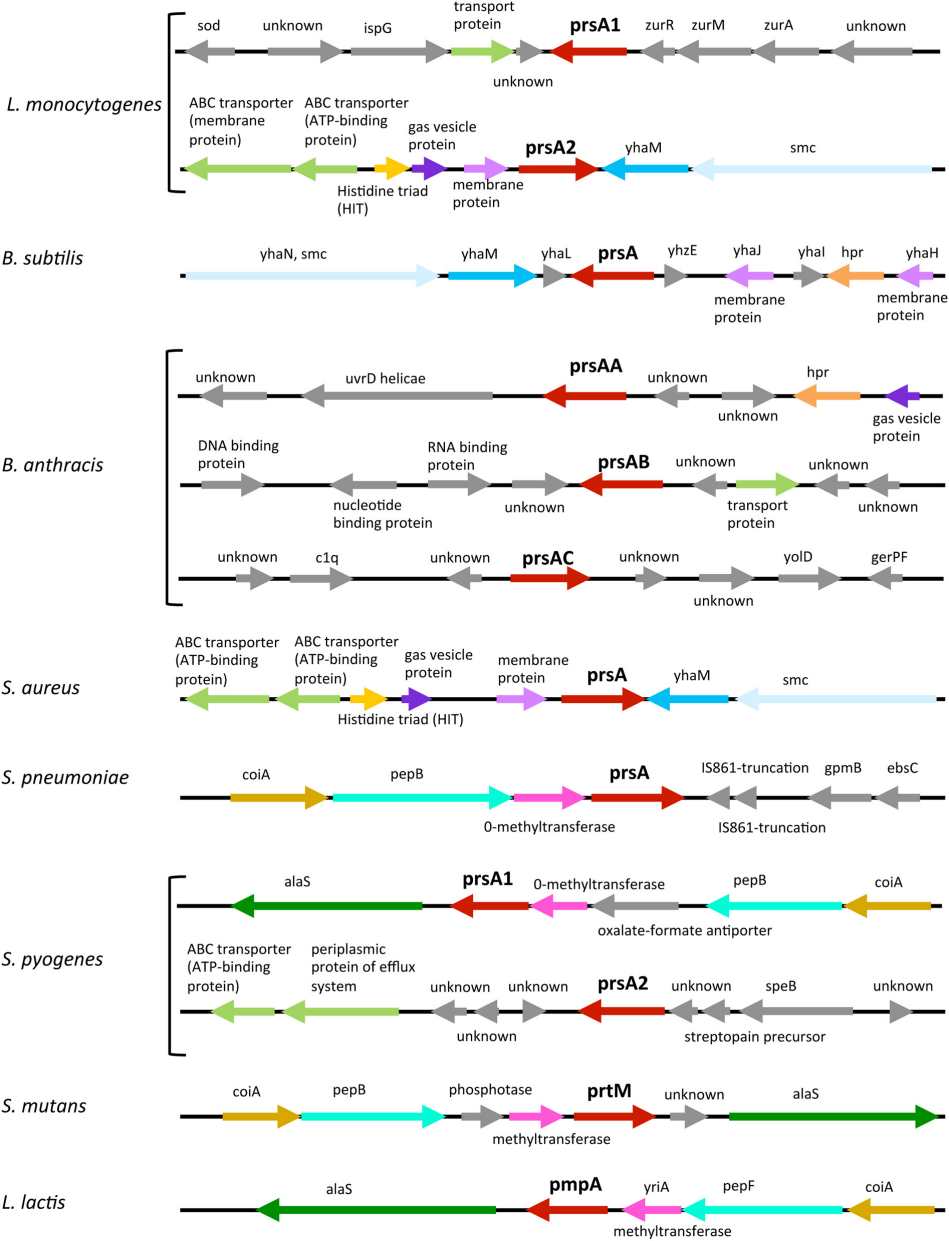
**Comparative organization of *prsA*-like loci**. The *prsA*-like loci from eight Gram-positive bacteria are shown where arrows represent open- reading frames. Genes with similar functions have the same color while genes that have unrelated functions are in gray. The annotation of genes mentioned in the text: *yhaN* encodes a putative ATPase involved in DNA metabolism containing a chromosome partition domain (smc), *yhaM* encodes a 3'-5' exoribonuclease, *coiA* encodes a competence transcription factor, *pep* is a peptidase, *htr* is a transcriptional regulator, and *alaS* is a alanyl-tRNA synthetase.

Given the critical requirement of PrsA2 for *L. monocytogenes* pathogenesis, how does it functionally compare with homologs expressed in other Gram-positive bacteria? *prsA*-like genes can be identified based on encoded amino acid sequence homology in many Gram-positive bacterial species (Figure 1). Thus far, the proteins have been best characterized in *L. monocytogenes* and *Bacillus subtilis*, although relatively little is still known about their molecular function. Interestingly, not all Gram-positive bacteria have the same number of PrsA-like proteins (Figure 2). *L. monocytogenes* has two, PrsA1 and PrsA2. As mentioned above, the function of *L. monocytogenes* PrsA1 has yet to be determined as deletion of the gene has yielded no discernable phenotype (Alonzo et al., 2009, 2011; Alonzo and Freitag, 2010). The expression of *prsA1* from the *prsA2* promoter does not complement a *prsA2* deletion, indicating that the lack of functional complementation observed was not due to differences in the timing of gene expression (Alonzo and Freitag, 2010). *Streptococcus pyogenes*, similar to *L. monocytogenes*, has two *prsA* alleles that also appear to encode proteins with non-redundant functions (Ma et al., 2006), whereas the related Streptococcal species *Streptococcus pneumonia* and *Streptococcus mutans* have only one *prsA* allele (Drouault et al., 2002; Guo et al., 2013). *B. subtilis* contains one *prsA* gene that was at first considered essential for viability but has recently been shown to be non-essential in the presence of high concentrations of magnesium (Hyyrylainen et al., 2010). In contrast, *Bacillus anthracis* has three *prsA* alleles that can complement *B. subtilis* PrsA, however whether one or more of these alleles are essential for *B. anthracis* viability is unknown (Williams et al., 2003). The single *prsA* alleles of *S. mutans*, *Staphylococcus aureus*, and *Lactococcus lactis* have been shown to be non-essential (Drouault et al., 2002; Jousselin et al., 2012; Guo et al., 2013).

Interestingly, there are some common themes with respect to the genomic organization of *prsA* alleles. *L. monocytogenes prsA2* and *S. aureus prsA* are most similar and are each adjacent to the 3′-5′ exoribonuclease YhaM coding sequence, as well as a gene encoding a putative ATPase involved in DNA metabolism (*smc*), and near an enzyme that acts as a nucleotidylyl hydrolase or transferase and ABC transporter genes (Figure 2). *B. subtilis prsA* is also located near *yhaM* (Figure 2). An alternative type of gene clustering is observed for bacteria for which the *prsA* allele is adjacent to methyltransferase and peptidase genes, such as the clustering found for *S. pneumoniae prsA* with the addition of an alanyl-tRNA synthetase gene for *S. pyogenes prsA1*, *S. mutans prtM*, and *L. lactis pmpA* (Figure 2). Organization of the *L. monocytogenes prsA1* locus is distinct from the loci mentioned above and does not appear to share many commonalities with the other *prsA* allele loci with the exception of being adjacent to a gene encoding a predicted transport protein (Figure 2).

## Shared family traits: PrsA2 functional domain organization

**Figure 3:**
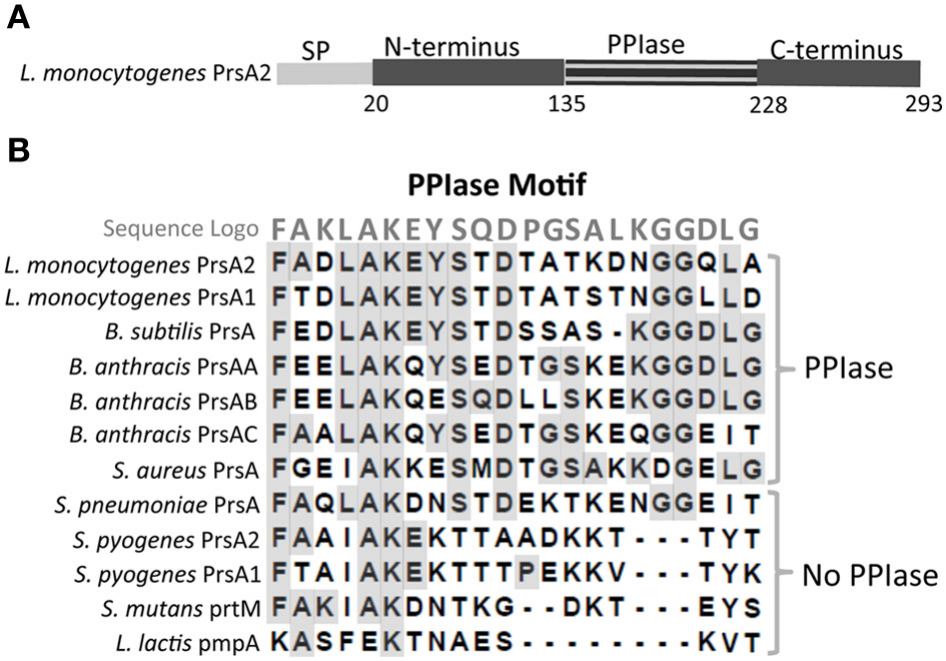
***L. monocytogenes* PrsA2 domain organization and the comparative alignment of PPIase domains of Gram-positive bacteria**. **(A)** Shown are the N-terminal signal peptide, N-terminus, PPIase domain, and C-terminus where numbers designate amino acids. **(B)** An alignment of the PPIase domain of 12 PrsA-like homologs is shown. The sequence logo is also indicated where identical amino acids are highlighted in gray. Homologs having a PPIase or no PPIase motif are designated.

*L. monocytogenes* PrsA2 is a lipoprotein and a member of a more extended and diverse class of chaperones known as parvulin peptidyl-prolyl isomerases (PPIases) that catalyze the *cis-trans* isomerization of peptide bonds N-terminal to proline residues in polypeptide chains (Alonzo et al., 2011). *L. monocytogenes* PrsA2 possesses a signal peptide that directs protein translocation across the bacterial cytoplasmic membrane via the Sec secretion pathway, an N-terminus and C-terminus that comprise the chaperone-like regions, and the central PPIase domain (Figure 3A). The *L. monocytogenes* PPIase domain contains a signature motif shared amongst PPIase containing proteins (Sigrist et al., 2010) (Figure 3B); however, not all PrsA-like proteins contain this PPIase motif. *L. lactis pmpA*, *S. pyogenes prsA1* and *prsA2*, and *S. mutans prtM* encoded proteins all lack the signature PPIase motif (Drouault et al., 2002; Guo et al., 2013) while *L. monocytogenes prsA1* and *prsA2*, *B. subtilis prsA*, *B. anthracis prsAA*, *prsAB*, and *prsAC* and *S. aureus prsA*, *and S. pneumoniae prsA* encode proteins with the centrally located motif (Williams et al., 2003; Vitikainen et al., 2004; Heikkinen et al., 2009; Alonzo et al., 2011) (Figure 3B). A functional PPIase domain has been shown using *in vitro* assays for *L. monocytogenes* PrsA2 and PrsA1 as well as for *B. subtilis* PrsA (Vitikainen et al., 2004; Tossavainen et al., 2006; Alonzo et al., 2011). The structural differences between the PrsA-like proteins suggests they may have two distinct and separable functions, N- and C-terminal foldase activity that is conserved among family members and PPIase activity that appears to be less highly conserved.

In Gram-positive bacteria, PrsA-like proteins have an N-terminal cysteine that upon signal sequence cleavage is modified with a diacylglycerol residue by the enzyme Lgt to promote membrane association (Leskela et al., 1999; Stoll et al., 2005; Baumgartner et al., 2007). In *L. monocytogenes*, *B. subtilis*, and *S. aureus*, disruption of *lgt* results in the increased release of PrsA2/PrsA and other lipoproteins into culture supernatants (Leskela et al., 1999; Stoll et al., 2005; Baumgartner et al., 2007). However, while *L. monocytogenes* PrsA2 is lipid modified and is membrane-associated, a significant amount of the protein is also secreted into culture supernatants (Alonzo et al., 2009). It remains to be determined whether both forms of *L. monocytogenes* PrsA2 (membrane-associated and secreted) are important for activity, and whether they fulfill distinct functions.

*L. monocytogenes* provides several advantages for studies focused on the molecular mechanisms underlying PrsA2 activity and its functional domains. For example, the bacterium is very amenable to genetic manipulation, thereby facilitating the demonstration that neither PrsA2 nor PrsA1 is essential given the fact that both genes can be deleted without deleterious effects to bacterial growth in broth culture (Alonzo and Freitag, 2010). Multiple assays can be used to assess the activity of PrsA2-dependent virulence factors *in vitro*, with the added dimension of tissue and animal culture models of infection. N- and C-terminal domain swap experiments between *L. monocytogenes* PrsA1 and PrsA2 in conjunction with site-directed mutagenesis experiments have indicated that PrsA2 tolerates substantial modifications without loss of activity. Data suggest that PrsA2 is likely to make multiple contacts with substrate proteins such that minor amino acid substitutions do not generally compromise protein function or substrate specificity (Alonzo et al., 2011). In addition, *L. monocytogenes ΔprsA2* mutants complemented with a construct encoding an N-and C-terminal PrsA2 fusion protein that completely lacks the central PPIase domain exhibited wild type levels of secreted hemolysin and phospholipase activities and formed normal plaques in fibroblast cell monolayers (Alonzo et al., 2011). These studies indicate that the PPIase domain is dispensable for some PrsA2-associated activities, however the complemented mutant had altered patterns of protein secretion, exhibited increased sensitivity to penicillin, and was defective in mouse models of infection (Alonzo et al., 2011).

## Signaling for a chaperone: regulation of PrsA expression

*prsA2* was initially identified as a result of its increased transcription following PrfA activation (Chatterjee et al., 2006). Subsequent studies confirmed that increased amounts of PrsA2 are synthesized and secreted in constitutively activated PrfA^*^ mutants (Port and Freitag, 2007), which led to the speculation that increased virulence factor secretion occurring as a result of PrfA activation requires the presence of additional amounts of chaperone to promote folding and activity and avoid membrane stress (Alonzo et al., 2009). In the absence of PrfA activation, *prsA2* reporter gene fusions indicate that *prsA2* expression increases during exponential growth and decreases upon entry into stationary phase (Forster et al., 2011). Whether other signals contribute to the regulation of *prsA2* expression is not known, and still less is known regarding the regulation of expression or function of PrsA1 in *L. monocytogenes*. Similarly, limited information is currently available regarding the regulation of PrsA-like homologs in other bacteria. In *S. pyogenes* the expression of *prsA2* appears to be negatively regulated either directly or indirectly by MtsR (metal transporter of streptococcus regulator) (Olsen et al., 2010). Disruption of *mtsR* increased the expression of *prsA2* in addition to altering transcript levels of multiple operons (Olsen et al., 2010). In *S. aureus*, *prsA* expression is directly regulated by the VraRS two-component sentinel system of cell wall stress (Jousselin et al., 2012). A VraR binding site was identified 84 base pairs upstream of the *S. aureus prsA* and deletion of *vraRS* caused a significant decrease in *prsA* basal mRNA levels (Jousselin et al., 2012). In addition, the VraRS two component regulator was triggered by cell wall active antibiotics vancomycin and teicoplanin which increased the expression of *prsA* (Jousselin et al., 2012). Lastly, in *L. lactis* some evidence suggests that nitrogen source may regulate *pmpA* expression but a regulator has not been identified (Drouault et al., 2002). *prsA* expression in Gram-positive bacteria thus appears to be regulated in response to different external stimuli. In the case of *L. monocytogenes* and *S. pyogenes*, each of which has two *prsA* alleles, only the regulation of *prsA2* has been observed.

## Deciding who needs a chaperone: identifying PrsA2 substrates

Studies in *B. subtilis* first identified a role for PrsA as a post-translocation secretion chaperone located at the membrane-cell wall interface (Kontinen and Sarvas, 1993). Because *prsA* initially appeared essential for *B. subtilis* viability, the influence of PrsA on patterns of protein secretion and folding were assessed using IPTG-inducible *prsA* constructs and monitoring the membrane and extracellular proteome of PrsA depleted cells compared to PrsA replete cells (Kontinen and Sarvas, 1993; Hyyrylainen et al., 2000; Wahlstrom et al., 2003; Vitikainen et al., 2004). Depletion of *B. subtilis* PrsA results in the accumulation of misfolded proteins at the cell envelope and triggers a membrane stress response (Hyyrylainen et al., 2001). The identification of non-essential PrsA2 in *L. monocytogenes* and the regulation of *prsA2* expression following PrfA activation enabled comparison of secreted protein profiles in Δ*prsA2*, wild type, and constitutively activated prfA^*^ strains (Alonzo and Freitag, 2010). Currently, many direct and/or indirect PrsA-like substrates have been identified in *L. monocytogenes* and *B. subtilis* through proteomic analyses (Milohanic et al., 2003; Alonzo and Freitag, 2010; Hyyrylainen et al., 2010). These studies suggest that both *L. monocytogenes* and *B. subtilis* share many direct and/or indirect PrsA2/PrsA substrates with roles in cell wall metabolism (penicillin binding proteins), swimming motility (flagellin) and chemotaxis, oligopeptide transport (OppA), as well as a quinol oxidase associated with membrane bioenergetics, surface-localized enolase, and secreted superoxide dismutase (Milohanic et al., 2003; Alonzo and Freitag, 2010; Hyyrylainen et al., 2010) (Table 1). The fact that *L. monocytogenes* PrsA2 and *B. subtilis* PrsA appear to share common substrates suggests that these two PrsA-like proteins have conserved foldase and PPIase functions. However, not all potential substrates are shared between the two organisms. *B. subtilis* PrsA has been associated with the folding and activity of alpha-amylase, an enzyme which hydrolyses starch to generate glucose, and subtilisin, an alkaline protease (Jacobs et al., 1993); *L. monocytogenes* does not secrete these enzymes. In contrast, *L. monocytogenes* PrsA2 has been linked to the folding and activity of several *L. monocytogenes* secreted virulence factors not expressed by *B. subtilis* (Alonzo and Freitag, 2010). Evidence suggests, for example, that loss of PrsA2 leads to improper folding and degradation of secreted pore forming toxin listeriolysin O (LLO) as indicated by Western blot analyses (Alonzo et al., 2009; Zemansky et al., 2009).

**Table 1:** **Potential PrsA2/PrsA substrates relevant to bacterial cell physiology**.

**Bacteria**	**PrsA-like cell effector**	**Gene function**	**References**
*Listeria monocy-*	Lmo2522	LysM domain penicillin binding protein	Alonzo and Freitag, 2010
*togenes*	PbpA	Penicillin binding protein 2a transpeptidase	Alonzo and Freitag, 2010
	PbpB	Penicillin binding protein 2b transpeptidase	Alonzo and Freitag, 2010
	Lmo0540	Penicillin binding protein, putative	Alonzo and Freitag, 2010
	OppA	Oligopeptide ABC-transporter, oligopeptide-binding protein	Alonzo and Freitag, 2010
	FlaA	Flagellin	Alonzo and Freitag, 2010
	QoxA	Quinol oxidase subunit II	Alonzo and Freitag, 2010
	Eno	Enolase	Alonzo and Freitag, 2010
	Sod	Superoxide dismutase	Alonzo and Freitag, 2010
*Bacillus subtilis*	AmyQ	α-amylase-hydrolyses starch to generate glucose	Kontinen and Sarvas, 1993
	AprE	Subtilisin- alkaline protease	Kontinen and Sarvas, 1993
	PbpA	Penicillin binding protein 2a transpeptidase	Hyyrylainen et al., 2010
	PbpB	Penicillin binding protein 2b transpeptidase	Hyyrylainen et al., 2010
	PbpC	Penicillin binding protein 3 transpeptidase	Hyyrylainen et al., 2010
	PbpD	Penicillin binding protein 4 transpeptidase	Hyyrylainen et al., 2010
	YvrA	ABC transporter ATP-binding protein	Hyyrylainen et al., 2010
	OppA	Oligopeptide-binding protein oppA precursor	Hyyrylainen et al., 2010
	FliF	Flagellar M-ring protein	Hyyrylainen et al., 2010
	FlhA	Flagellar biosynthesis protein flhA	Hyyrylainen et al., 2010
	YlxF	FlaA locus 22.9 kDa protein	Hyyrylainen et al., 2010
	QoxB	Quinol oxidase subunit I	Hyyrylainen et al., 2010
	Eno	Enolase	Vitikainen et al., 2004
	SodA	Superoxide dismutase	Vitikainen et al., 2004

Activation of the central virulence regulator PrfA results in increased expression of a number of secreted proteins that play central roles in *L. monocytogenes* pathogenesis (Leimeister-Wachter et al., 1990; Mengaud et al., 1991; Port and Freitag, 2007; Scortti et al., 2007; de Las Heras et al., 2011). These include the internalins InlA and InlB, associated with bacterial entry into host cells (Gaillard et al., 1991; Lingnau et al., 1995), LLO (Gaillard et al., 1987; Kuhn et al., 1988; Bielecki et al., 1990), the phospholipases PlcA and PlcB which mediate vacuole membrane lysis (Marquis et al., 1995; Smith et al., 1995; Grundling et al., 2003), the surface protein ActA which mediates actin polymerization (Domann et al., 1992; Vazquez-Boland et al., 1992; Brundage et al., 1993; Sokolovic et al., 1993), InlC which reduces cortical tension to facilitate bacterial cell-to-cell spread with in the host (Engelbrecht et al., 1996; Rajabian et al., 2009), and PrsA2 (Port and Freitag, 2007; Alonzo et al., 2009). Comparisons of the secreted protein profiles between *L. monocytogenes* wild type and strains lacking PrsA2 or containing constitutively activated PrfA suggests that PrsA2 contributes to the folding and activity of a number of PrfA-regulated gene products, including LLO, ActA, PlcB, InlC, and the metalloprotease that processes pro-PlcB to its activated form, Mpl (Alonzo and Freitag, 2010; Forster et al., 2011). Additional gene products that contribute to *L. monocytogenes* pathogenesis are associated with PrsA2 activity; these gene products do not appear to be directly regulated by PrfA but their secretion increases as a result of PrfA activation (Alonzo and Freitag, 2010). These include CtaP, a cysteine-transport associated lipoprotein associated with host cell adhesion, acid resistance, and bacterial membrane integrity (Xayarath et al., 2009), and ChiA, a chitinase that inhibits the expression of host inducible nitric oxide synthase (iNOS) (Chaudhuri et al., 2013). Both of these proteins have been identified as potential substrates of PrsA2 via proteomic analysis (Alonzo and Freitag, 2010). In the absence of *prsA2*, PrfA activation reduces *L. monocytogenes* fitness and compromises bacterial membrane integrity (Alonzo and Freitag, 2010). Taken together, these findings suggest that PrsA2 has evolved to promote the folding and activity of virulence factors whose expression is increased as a result of PrfA activation within mammalian host cells.

PrsA-like proteins have been associated with virulence factor secretion and activity in other Gram-positive bacterial pathogens. *S. pyogenes* PrsA2 is required for production of the fully mature, enzymatically active streptococcal cysteine protease SpeB (Ma et al., 2006) and loss of PrsA2 and thus SpeB activity has been associated with a reduction in the ability of *S. pyogenes* to cause necrotizing fasciitis (Olsen et al., 2010). In *B. anthracis*, production of protective antigen PA, a component of lethal and edema toxins, is dependent on PrsA (Williams et al., 2003). As we gain a better understanding of the activities of PrsA chaperones and how substrate recognition occurs, it is likely that the list of secreted proteins that depend upon PrsA-like molecules for folding and activity will increase in number.

## Building a better bacterium: the role of PrsA-like homologs in cell wall biosynthesis and cell physiology

While evidence indicates that PrsA2 plays a critical role in the folding and stabilization of *L. monocytogenes* secreted virulence factors, PrsA2 has additional roles through its contributions to cell wall biosynthesis, resistance to osmotic stress, and swimming motility. *L. monocytogenes* strains lacking *prsA2* exhibit increased sensitivity to penicillin, which targets peptidoglycan transpeptidation (Alonzo et al., 2011), and are also more sensitive to incubation in the presence of lysozyme (Forster et al., 2011). Strains that lack *prsA2* have reduced levels of several penicillin binding proteins (PBPs) as well as cell wall modifying enzymes (Alonzo and Freitag, 2010), suggesting that selected aspects of *L. monocytogenes* cell wall biosynthesis may be altered in Δ*prsA2* strains. Interestingly, PrsA2 mutants that lack the central PPIase domain but retain the N- and C-terminal foldase domains exhibit normal patterns of secretion and activity for a number of potential substrate proteins, including LLO and PlcB, but remain sensitive to penicillin (Alonzo et al., 2011). This finding suggests that PrsA2 substrates may be of two classes: those that require foldase function without PPIase activity, and those dependent on PPIase. Several PrsA2-associated PBPs have significant numbers of proline residues (ranging from 2 to 4% of the total amino acids), and thus may require PPIase activity for proper folding.

Evidence for PrsA-like protein contributions to cell wall biosynthesis and integrity in other bacteria is accumulating. In *B. subtilis*, PrsA depleted cells show a decrease in the degree of peptidoglycan cross-linkages (Hyyrylainen et al., 2010). In *S. mutans*, physical cell wall integrity of the *prsA* mutant was tested with sonication and revealed a drastic reduction in viability when compared to the wild-type strain (Guo et al., 2013). Disruption of *prsA* in *S. aureus* was found to cause bacterial sensitivity to both glycopeptides and oxacillin (Jousselin et al., 2012). In *L. lactis*, the inactivation of the *prsA*-like gene, *pmpA* caused increased sensitivity to ionic and non-ionic osmotic shock (Drouault et al., 2002). In addition in *L. lactis*, inactivation of *pmpA* caused a 3-fold decrease in growth rate when exposed to ionic (0.25 M NaCl) or non-ionic (0.6 M sorbitol) shock (Drouault et al., 2002). These *prsA*-associated phenotypes may be a direct result of a compromised cell wall and/or result from defects in the folding and activity of membrane and cell wall proteins associated with resistance to osmotic stress.

Another potential PrsA2/PrsA substrate identified in *L. monocytogenes* and *B. subtilis* was flagellin, required for the assembly of flagella and bacterial swimming motility (Alonzo and Freitag, 2010; Hyyrylainen et al., 2010). It has not yet been determined whether the FlaA subunit itself or alternatively some component(s) required for the assembly of the flagellar apparatus may require PrsA2 for folding. *L. monocytogenes* strains are flagellated and motile at low temperatures but have reduced motility at higher temperatures (37°C) associated with mammalian hosts (Peel et al., 1988). *L. monocytogenes* strains lacking *prsA2* exhibit significantly reduced swimming motility at both low and high temperatures (Zemansky et al., 2009). Thus, while PrsA2 is required for *L. monocytogenes* pathogenesis and full bacterial viability during bacterial replication within the cytosol, it additionally contributes to several important aspects of *L. monocytogenes* physiology (osmotic resistance and bacterial motility) during bacterial growth outside of the host.

## Putting it all together: modeling the functional contributions of PrsA2 to *L. monocytogenes* protein secretion both inside and outside of host cells

**Figure 4:**
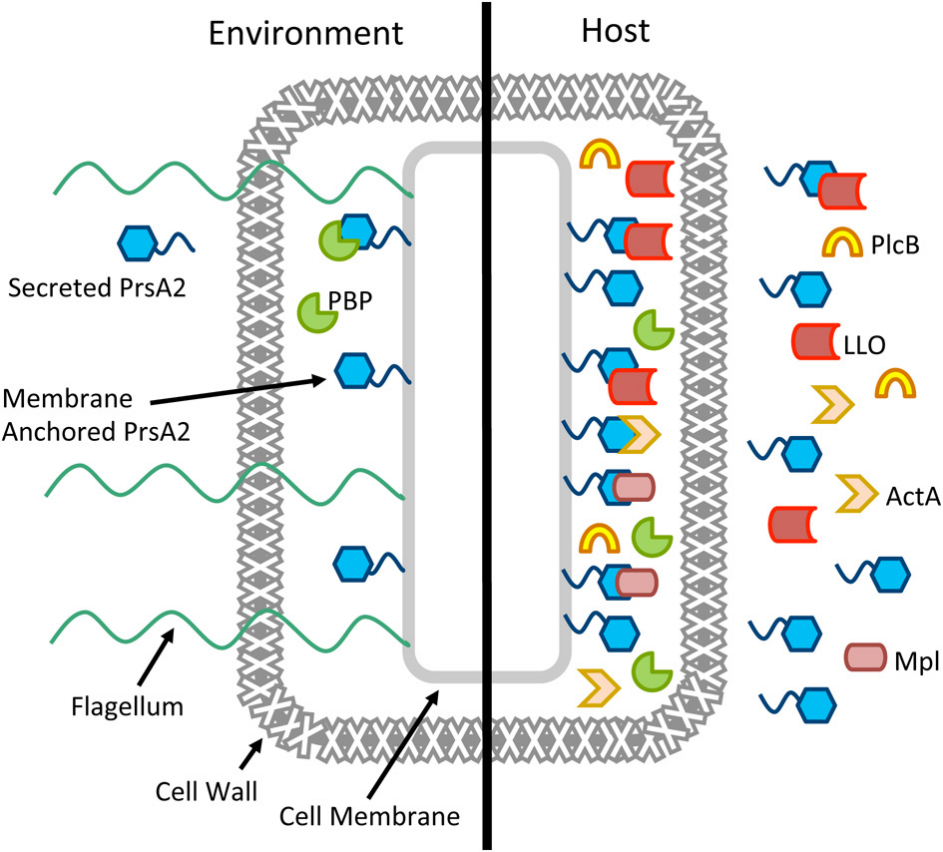
**Working model for the multiple roles of PrsA2 in *L. monocytogenes***. A cartoon of a single *L. monocytogenes* cell is shown in the environment and in the host, with PrsA2 both tethered to the cell membrane and secreted. When *L. monocytogenes* is in the outside environment, PrsA2 directly or indirectly is required for functional penicillin binding proteins (PBP), flagellin, and other factors that contribute to cell wall integrity, swimming motility, and resistance to osmotic stress. When *L. monocytogenes* is in the host, activation of the central virulence regulatory protein PrfA leads to increased expression of several secreted virulence factors as well as PrsA2, which is required for the folding and stability of listeriolysin (LLO), the Mpl protease that activates the broad-range phospholipase (PlcB), ActA, and other factors.

In Gram-positive bacteria, most secreted proteins are thought to cross the single bacterial cell membrane in an unfolded state to enter the space that exists between the cell membrane and the cell wall (Matias and Beveridge, 2005, 2006). This inner wall space characteristically contains high concentrations of cations bound to teichoic acid, a high density of negative charge, and a low pH which presents a challenging environment for protein folding and function (Sarvas et al., 2004). The proposed function of *L. monocytogenes* PrsA2 and related PrsA-like proteins in other Gram-positive bacteria is to promote post-membrane translocation protein folding and maintain optimal secretion homeostasis. Although *L. monocytogenes* PrsA2 contributes to several aspects of *L. monocytogenes* physiology during bacterial growth in broth culture or on solid media, its most striking contributions are detected during bacterial infection of mammalian cells where it is essential for the folding and activity of secreted proteins required for bacterial virulence and intracellular bacterial replication (Alonzo et al., 2009, 2011; Zemansky et al., 2009; Alonzo and Freitag, 2010) (Figure 4).

During bacterial growth in rich media, *prsA2* is expressed at a basal level with maximal expression occurring during exponential growth; expression rapidly decreases upon entry into stationary phase (Forster et al., 2011). PrsA2 associates with the bacterial membrane presumably as a result of the diacylglycerol modification of its N-terminal cysteine by the enzyme Lgt (Baumgartner et al., 2007). A significant fraction of PrsA2 can also be detected in bacterial supernatants; interestingly, secreted PrsA2 still appears to retain the N-terminal lipid modification (Baumgartner et al., 2007). PrsA2 assists indirectly or directly in the proper folding of substrates required for swimming motility, peptidoglycan synthesis, and osmotic stability (Zemansky et al., 2009; Alonzo and Freitag, 2010; Forster et al., 2011). Secretion of a second PrsA-like protein, PrsA1, has been detected for *L. monocytogenes* grown in broth culture and experimental evidence suggests that the protein is functional based on its PPIase activity (Alonzo et al., 2011), but the substrates for this chaperone have not been identified and its functional significance is not yet known.

Following *L. monocytogenes* entry into host cells, the virulence regulator PrfA becomes activated and increases gene expression by binding to target promoters that contain a PrfA palindromic DNA binding site (or PrfA box) (Xayarath and Freitag, 2012). PrfA activation leads to increased expression and secretion of a variety of protein products associated with bacterial virulence, including PrsA2 (Port and Freitag, 2007). The PrfA-dependent increase in secreted PrsA2 appears necessary for: (1) the proper folding and stability of several secreted virulence factors, including LLO, Mpl, and PlcB; (2) the maintenance of bacterial membrane integrity; and (3) the proper biosynthesis and modulation of the bacterial cell wall (Figure 4). *L. monocytogenes* strains lacking PrsA2 exhibit reduced viability under conditions of PrfA activation and are severely attenuated for virulence in mice (Alonzo et al., 2009, 2011; Alonzo and Freitag, 2010).

What are the critical aspects of PrsA2 function that promote *L. monocytogenes* survival and replication within the infected host? Mutants lacking PrsA2 and the secreted chaperone-protease HtrA rapidly lose viability under conditions of PrfA activation, suggesting that the loss of foldase activity and presumably HtrA protease degradation of misfolded proteins leads to a membrane stress response that reduces bacterial fitness within the cytosol (Alonzo and Freitag, 2010). It is possible that it is the reduction in bacterial fitness resulting from misfolded proteins and membrane stress, rather than a reduction in the secretion of a particular set of virulence factors, that leads to the dramatic 100,000-fold level of attenuation observed for Δ*prsA2* mutants in mice (Alonzo et al., 2009). Interestingly, evidence suggests that both the foldase activity and the PPIase activity are required for full virulence, as PrsA2 mutants that lack PPIase activity but retain foldase remain attenuated in mouse models of infection, albeit to a lesser degree than full deletion mutants (Alonzo et al., 2011).

There are many functional aspects of *L. monocytogenes* PrsA2 and PrsA-like proteins in other Gram-positive bacteria that remain unknown. As yet, no structural information is available for this class of chaperones other than the reported tendency of *L. monocytogenes* PrsA2 and *B. subtilis* PrsA to form dimers (Hyyrylainen et al., 2010; Alonzo et al., 2011). How PrsA2 recognizes and interacts with its target substrates remains to be determined, as does the active form(s) of the protein (membrane associated vs. secreted). It is possible that the membrane-associated and secreted forms of PrsA2 serve distinct functions, such that the membrane-associated form is required for the correct folding of proteins following membrane translocation, while the secreted form may remain bound to the folded substrate to prevent aggregation or degradation until substrate activity is required. A more detailed understanding of PrsA2 and related homologs will not only increase current understanding of the process of protein secretion in Gram-positive bacteria, but may yield new strategies for the optimization of active protein secretion for purification or foreign antigen secretion in Gram-positive bacteria-based vaccine vectors. PrsA-like proteins may also be effective targets for small molecule inhibitors that could potentially reduce the production of secreted virulence factors during host infection. Given these possibilities, PrsA2 and PrsA-like proteins are chaperones whose molecular résumés are definitely worth a detailed look.

## Authors and contributions

Laty A. Cahoon and Nancy E. Freitag contributed to the conception and writing of this review.

### Conflict of interest statement

The authors declare that the research was conducted in the absence of any commercial or financial relationships that could be construed as a potential conflict of interest.
